# Joubert syndrome; misleading presentation of two cases as pseudo-tumor cerebri and literature review

**DOI:** 10.15171/jrip.2017.14

**Published:** 2016-11-07

**Authors:** Farrokh Seylanian Toosi, Samineh Boloursaz, Bita Abbasi, Reza Hekmat, Reihaneh Mortazavi Ardestani, Mina Mohajerzadeh

**Affiliations:** ^1^Department of Radiology, Mashhad University of Medical Sciences, Mashhad, Iran; ^2^Department of Nephrology, Mashhad University of Medical Sciences, Mashhad, Iran

**Keywords:** Joubert syndrome, Renal transplantation, Retinitis pigmentosa

## Abstract

Joubert syndrome is a rare autosomal recessive disorder that may have different clinical presentation such as ataxia, hyperpnea, sleep apnea, nystagmus, hypotonia, seizure and retinitis pigmentosa. We present a 22-year-old girl and her older sibling, labeled as cerebral palsy. She had renal transplant years ago without the true diagnosis of the disorder. Brain imaging revealed the classic "molar tooth sign" appearance, and clinical evaluation established the diagnosis for both of the siblings. Imaging should be done to evaluate the neuroradiological findings of Joubert syndrome. With a neonate with Joubert syndrome in a family, antenatal diagnosis by ultrasound is crucial for future siblings.

**Implication for health policy/practice/research/medical education:**

Joubert syndrome is a rare genetic disorder. It is characterized by nephrologic, neurologic and ophthalmologic disorders. Absence or underdevelopment of the cerebellar vermis, malformed brain stem (molar tooth sign), ataxia, hyperpnea, sleep apnea, nystagmus, hypotonia and seizure are the neurologic features of this syndrome. It is one of the causes of retinitis pigmentosa syndrome. The most common nephrologic presentations of Joubert syndrome are cystic dysplasia and nephronophthisis.

## Introduction

Joubert syndrome and related disorders are known as a group of developmental delay and several congenital anomalies (1). Its incidence is estimated between 1/80 000 to 1/100 000 live birth (2). Three classic characteristic features of Joubert syndrome are hypotonia, developmental delay and cerebellar malformation. Other findings are breathing abnormalities and truncal ataxia that improve over time (1).

Conditions related to Joubert syndrome include polydactyly, ocular coloboma, retinal dystrophy, renal disease including cystic dysplasia or nephronophthisis and hepatic fibrosis. It is important to know some of them are not apparent at birth (2,3). In a brief review of neuropathology, the most prominent feature is the absence of cerebellar vermis. Patients suffer from dysregulation of muscle tone, abnormal saccadic eye movement and truncal ataxia. Several malformations of pons and medulla are responsible for respiratory disorders such as reticular formation, basis pontis and inferior olivary. Purkinje-like neuron heterotopias can explain symptoms. Absence of superior cerebellar peduncle and corticospinal tract decussation at medullary pyramids displays altered brain wiring (4,5).

A broad spectrum of ophthalmologic findings are noted. Nystagmus, dysconjugate gaze, oculomotor apraxia, amblyopia, ptosis, and optic disc drusen have been observed. There are two basic forms of retinal disease: severe congenital blindness known as Leber congenital amaurosis and a latter-onset pigmentary retinopathy (5). In the group with the retinal dystrophy prevalence of cystic renal disease appears to be higher and worse prognosis is predicted (1).

Liver involvement is the prominent feature of COACH syndrome, presents with portal hypertension or elevated serum transaminases. Radiologically, increased echogenicity and cyst may be visible on liver ultrasound, and dilated intrahepatic bile duct on liver magnetic resonance imaging (MRI) (5).

Other manifestations include renal disease, hepatic involvement and skeletal features. Prevalence of renal disease estimated up to 30% of subjects and presents in two forms. First cystic dysplasia is identified prenatally by ultrasound characteristic of Dekaban-Arima syndrome. The other more common is juvenile nephronophthisis characterized by tubulointerstitial nephritis and cyst concentrated at the corticomedullary junction. Progression to end-stage renal disease occurs by approximately 13 years old. Ultrasound changes include increased renal echogenicity with small, scarred kidneys (5).

## Case Presentation

**Figure 1:**
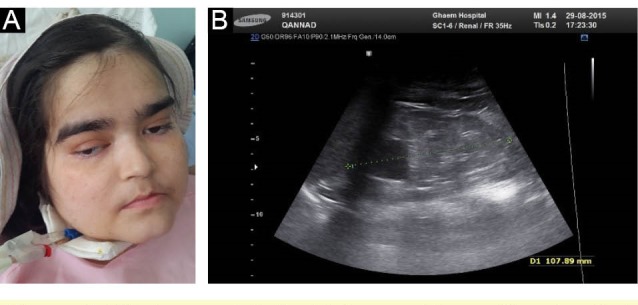
(A) shows subtle facial dysmorphism, with epicanthal fold, hypertelorism, oculomotor apraxia, and actually right frozen eye you can also see jugular catheter for temporary use of urgent dialysis. (B) sonographic appearance of a normal sized echogenic kidney with cystic changes in corticomedulary junction.

**Figure 2:**
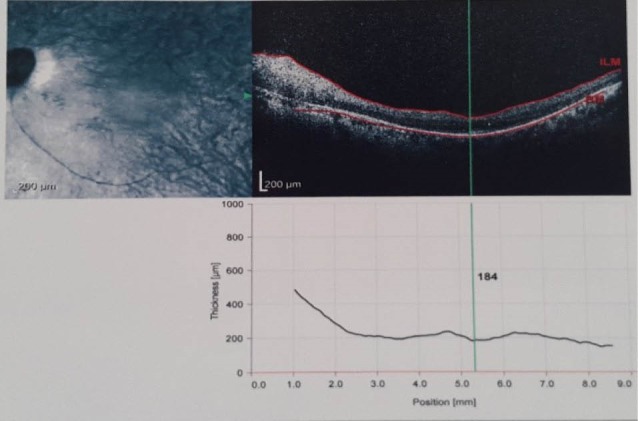
Macular optical coherence tomography shows diffuse retinal atrophy in both eyes, documenting retinitis pigmentosa.

**Figure 3:**
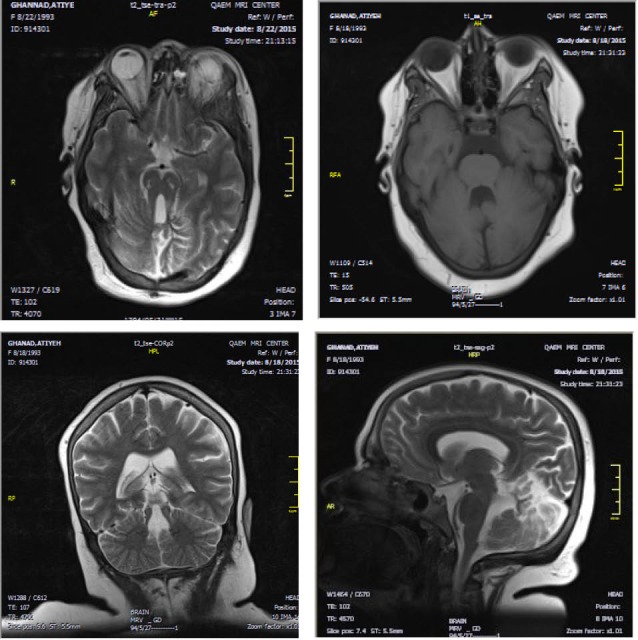
MRI appearance of patient. (A) T2W axial image of brain stem shows deep interpeduncular fossa, thickening and elongation of the superior cerebellar peduncle with horizontal orientation giving “molar tooth sign.” (B) T2W coronal shows hypoplastic vermis. (C) T1W axial shows batwing shaped fourth ventricle. (D) T2W vermis hypoplasia involves both the superior and inferior vermis with an open communication between the fourth ventricle and the extra cerebellar subarachnoid space. Cerebellar hemispheres in contact with the midline.is also visualized as a result of severe vermis hypoplasia.

We present a 22-year-old girl and her older sibling, labeled as hypotonic cerebral palsy since the time they were born. Developmental delay, mental retardation, hypotonia, ataxia, obesity, respiratory and ophthalmological disorders, facial dysmorphism, were the other clinical findings. Hypotonia and ataxia were accompanied with normal deep tendon reflexes and negative babinski sign. She showed facial dysmorphism with epicanthal fold (Figure 1A) and frontal bossing without any sign of anomalous dentition or cleft lip or palate. Tongue protrusion was seen to some degree. She had convulsive attack in acute phase of renal failure that could not be discriminated whether her neurologic disorders or metabolic effect of renal dysfunction were responsible. She had ophthalmological findings such as epicanthal fold, hypertelorism, oculomotor apraxia and nystagmus. We also found waxy pallor of disc, arterial narrowing and bone spicule in fundoscopy, compatible with “retinitis pigmentosa.” Macular optical coherence tomography was done and shows diffuse retinal atrophy (Figure 2). According to mental retardation ophthalmological examination was always difficult, however, it was conducted at the end stage of disease with a waxy pallor of optic disc and degrees of retinal atrophy. Patient had renal transplantation 13 years ago. Renal sonographic appearance was the normal size kidneys with echogenic parenchyma and multiple cysts in corticomedullary junction (Figure 1B). The striking features of this patient were that funduscopic findings in acute phase of renal transplant rejection, were related falsely to pseudo-tumor cerebri or cyclosporine effect. Liver enzymes were in normal range and we did not find any abnormal sonographic features. Electroencephalography was normal. Brain imaging revealed the typical nervous system malformation (Figure 3). There was cystic lesion seen in brain stem that we did not understand its clinical significance. According to clinical and radiological findings, Joubert syndrome with oculo-retinal disease or Dekaban-Arima syndrome was our diagnosis.

**Figure 4:**
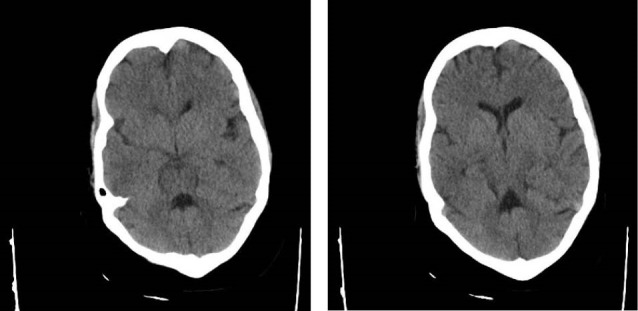
Axial CT-Scan molar tooth sign and batwing shaped forth ventricle from older sibling

Patient was born from consanguine parents with the history of an older sibling, who died with the same presentation. Her older sibling had brain imaging that shows the typical manifestations of Joubert syndrome (Figure 4). He had the same presentation of hypotonia at birth that was labeled as hypotonic cerebral palsy, and the same course of renal insufficiency. He died two years ago because of renal failure without a definite diagnosis.

The patient was kept on supportive treatment which consisted of renal transplant management, neuropsychological support and physical rehabilitation.

## Discussion

After clinical suspicion of Joubert syndrome related disorders judging from hypotonia, developmental delay and abnormal breathing, a brain MRI for molar tooth sign (MTS) for the first step was suggested (2). Although the MTS is proposed as a hallmark feature of Joubert’s brain imaging, some other authors believe that it is not pathognomonic of Joubert syndrome and can be seen in other disorders including oral-facial-digital syndrome type VI (OFDVI ) (6). Hence, there is a general term describing all related anomalies including OFDVI termed Joubert syndrome related disorders (JSRD).

Brain imaging helps to establish the diagnosis. Hypoplastic vermis with a batwing shaped fourth ventricle, hypoplastic isthmus with a deep interpeduncular fossa, thinned pontomesencephalic junction, thickening and elongation of the superior cerebellar peduncle with horizontal orientation giving “molar tooth sign” is seen (7). Vermian hypoplasia involves both the superior and inferior vermis with an open communication between the fourth ventricle and the extracerebellar subarachnoid space. Cerebellar hemispheres in contact with the midline is also visualized as a result of severe vermian hypoplasia (1).

Association with corpus callosum dysgenesia (6%-10%), posterior fossa malformation, ventriculomegaly (due to atrophy in 6%-20%), and evidence of neuronal migration anomalies are other findings (6,7). Wide range of associated brain imaging abnormalities are also reported including cystic enlargement of posterior fossa, white matter cysts, neuroepithelial cysts, hypothalamic hamartoma, and absence of the pituitary gland (1). Abnormal migration defects, named in literature are mainly periventricular nodular heterotopia, and cortical organization defect such as polymicrogyria that cause epilepsy as a rare feature of Joubert syndrome (1,3). Occipital meningoencephalocele in also noted as a rare presentation (1,3). Cerebellar heterotopia and cerebellar folial disorganization and hypocampal malformation is reported (1,3). Cerebellar malformation is probable cause of ataxia and speech dyspraxia (3). Absence of decussation of the corticospinal tract and superior cerebellar tract based on diffusion tensor imaging, and abnormal activation patterns during motor tasks based on functional MRI studies are other investigations (3).

We should take into consideration the systemic metabolic disorders that can imitate the neurologic and ophthalmologic feature of JSRD (7).

Management should include physical and psychological support, management of breathing abnormalities and medication use (7). Some authors suggest monitoring renal function and perform ultrasonography of the kidneys to detect cystic renal disease for patients with retinal dystrophy (1). A neonate with Joubert syndrome in a family antenatal diagnosis by ultrasound in utero for future siblings is crucial (1). Whereas fetal brain neuroimaging may remain uninformative until the end of the second trimester of pregnancy (1).

## Conclusion

Joubert syndrome related disorders are groups of genetic disease. For any patient with ataxia, nystagmus, hypotonia and developmental delay it should be considered, and imaging should be done to evaluate neuroradiological findings. Molar tooth sign is the hallmark of Joubert syndrome. Hepatic, renal, and retinal involvement should be noted to slow down the natural history of this disorder.

## Authors’ contribution

All authors contributed to write the manuscript. FST, BA, RH, RMA and MM conducted data collection and case presentation. SB prepared the manuscript. All authors read, revised, and approved the final manuscript.

## Conflicts of interest

The authors declared no competing interests.

## Ethical considerations

Ethical issues (including plagiarism, data fabrication, double publication) have been completely observed by the authors.

## Funding/Support

None.
